# Haemolytic uremic syndrome surveillance in children less than 15 years in Belgium, 2009–2015

**DOI:** 10.1186/s13690-018-0289-x

**Published:** 2018-08-06

**Authors:** S. Jacquinet, K. De Rauw, D. Pierard, N. Godefroid, L. Collard, K. Van Hoeck, M. Sabbe

**Affiliations:** 1 Service of Epidemiology of Infectious Diseases, Department of Public Health and Surveillance, Sciensano, Brussels, Belgium; 2 Vrije Universiteit Brussel (VUB), Department of Microbiology and Infection Control, National Reference Centre for STEC, Universitair Ziekenhuis Brussel (UZ Brussel), Laarbeeklaan 101, 1090 Brussels, Belgium; 3 Service de néphrologie pédiatrique, Cliniques Universitaires Saint Luc, UCL, Brussels, Belgium; 4 Centre Hospitalier Chrétien, Liège et Centre Hospitalier Universitaire de Liège, Liège, Belgium; 5 Department Paediatrics Faculty Medicine and Health Science, Universiteit Antwerpen, Antwerpen, Belgium

**Keywords:** Haemolytic uremic syndrome, Shiga toxin-producing *Escherichia coli*, Surveillance, Belgium

## Abstract

**Background:**

The Haemolytic Uremic Syndrome (HUS) is the most severe manifestation of infection with Shiga toxin-producing *Escherichia coli* (STEC). In Belgium, the surveillance of paediatric HUS cases is conducted by a sentinel surveillance network of paediatricians called Pedisurv. In this article, we present the main findings of this surveillance from 2009 to 2015 and we describe an annual incidence of HUS.

**Methods:**

For each case of HUS <  15 years notified by the paediatricians, clinical, microbiological and epidemiological data were collected by a questionnaire. National hospital discharge data with ICD-9 code 283.11 were used to calculate the incidence of HUS in children < 15 years.

**Results:**

From 2009 to 2015, 110 cases were notified to the Pedisurv network with a mean annual notification rate of 0.8/100,000 in children < 15 years. Death occurred in 2.5% of all patients and the median number of days of hospitalization was 10 days. One third (35.4%) of the HUS cases were confirmed positive STEC, with a majority of STEC O157. The mean annual incidence based on the hospital discharge data was 3.2/100,000 in children < 15 years and 4.5/100,000 in children < 5 years.

**Conclusion:**

The incidence of paediatric HUS in Belgium is high compared to other European countries. Its surveillance in Belgium is quite comprehensive and, although less effective than monitoring all STEC infections to detect the emergence of outbreaks, is important to better monitor circulation of the most pathogenic STEC strains. In this context, efforts are still needed to send samples and STEC strains from HUS cases to the National Reference Centre.

## Background

The Haemolytic Uremic Syndrome (HUS), characterised by a triad of haemolytic anaemia, thrombocytopenia and acute renal failure, is the most severe manifestation of infection with Shiga toxin-producing *Escherichia coli* (STEC) [1]. HUS is the first cause of acute renal impairment in children under five years of age [1]. The case fatality rate ranges from 2 to 7%; whereas 12 to 30% of the cases remain with long term sequelae such as renal impairment, hypertension or neurological injury [2, 3].

Ninety percent of HUS in children are caused by STEC infections (also called STEC-HUS) [2] with STEC O157:H7 as the most frequent serotype responsible for HUS in Europe [4–6]. Non-HUS, less frequent, can be caused by *Streptococcus pneumoniae* infection or methyl-malonic aciduria or can be atypical HUS (aHUS). This form of HUS occurs at any age, and may be sporadic or familial and a clear link has been demonstrated with genetic abnormalities in complement regulatory genes [2, 7].

Surveillance of HUS is important to detect outbreaks, strains of STEC associated with severe outcomes, to detect the emergence of new strains of STEC and to find incriminated products [8, 9].

According to a multicenter study performed in Belgium in 1996, the incidence of HUS was 4.3/100,000 in children under 5 years and 1.8 /100,000 in children under 15 years [10], one of the highest reported in Western Europe [6].

In Belgium, HUS is monitored specifically by the sentinel surveillance network of paediatricians (Pedisurv). The National Reference Centre (NRC) for human STEC infections and the mandatory notification in the three regions of Belgium monitors the STEC infections. The objective of the monitoring via Pedisurv is to highlight the burden of disease of HUS on the Belgian children and collect epidemiological, clinical and microbiological data for cases of paediatric HUS.

In this article, we present the main findings of this surveillance from 2009 to 2015, and we describe an annual incidence of HUS based on this network and on hospital discharge data.

## Methods

The prospective surveillance of HUS in children of < 15 years of age started from 1st January, 2009 through the surveillance network called Pedisurv, with the support of the Belgian NRC for STEC. This report summarizes findings until 31st December 2015. The surveillance network, created in 2002 and coordinated by the Belgian Institute of Public Health, monitors several uncommon paediatric infectious diseases in children under 15. Currently, Pedisurv monitors the occurrence of acute flaccid paralysis, measles, mumps, invasive pneumococcal disease and congenital rubella. Paediatricians participate to the Pedisurv network on a voluntary basis with an average participation of 300 paediatricians per year (of which 190 are hospital based). The coverage of this network was only calculated for invasive pneumococcal disease (61% of Belgian paediatricians) but is unknown for HUS [11].

Paediatricians reported monthly how many new cases of HUS they had diagnosed. To avoid omissions, it was also asked to indicate if zero cases were encountered. A standard, structured questionnaire was completed by the reporting paediatricians to collect basic epidemiologic data that included clinical features, laboratory investigations, outcome and occurrence of other cases of HUS or diarrhoea in the community in the previous month. Exposure to potential risk factors in the 15 days before onset of HUS was also included in the questionnaire. Risk factors, such as consumption of unpasteurized dairy products and ground beef, contact with farm animals, and recreational exposure to water were considered.

Clinicians and laboratories are encouraged to send faecal samples, rectal swabs, serum samples or locally cultivated isolates of HUS patients to the NRC for diagnosis and confirmation of STEC infection. At the NRC all faecal samples and rectal swabs are cultured on sorbitol-MacConkey agar (SMAC) with and without tellurite and cefixime and after overnight incubation, the cultures are screened for the presence of Shiga toxin genes (*stx*) using PCR [12]. The presence of antibodies against the lipopolysaccharide (LPS) of the most frequent O-serogroups (O26, O103, O111, O121, O145, and O157) is determined in the serum samples by a micro-agglutination technique. All isolated strains are serotyped, additional virulence genes (*eaeA*, *ehxA*, *aaiC* and *aggR*) and *stx* subtypes are determined, and antimicrobial susceptibility testing is performed [12–14].

A case of paediatric HUS was defined as a child 0–14 years old with a clinical diagnosis of HUS: sudden onset of haemolytic anaemia (haemoglobin < 10 g/dl with/or fragmentocytes > 2%) with renal insufficiency (at least one of the following: elevated creatinemia (above normal values for age and sex), haematuria > 20,000/ml or proteinuria > 1.0 g/l) and thrombocytopenia (< 160,000 thrombocytes/mm^3^).

A case of STEC infection was confirmed by the isolation of STEC strains, by PCR detection of *stx* genes in the faecal cultures or by finding antibodies against LPS of one of the 6 serogroups of STEC tested.

A confirmed case of HUS was any person meeting the clinical criteria of HUS with a confirmed STEC infection.

The inclusion criteria for this study were: age between 0 and 14 years, being hospitalized in Belgium, recorded through Pedisurv between 1/01/2009 and 31/12/2015 and corresponding to the case definition. Duplicates were removed by using name initials, date of birth and postal code. Where duplicates were identified, the most complete questionnaires were used in preference.

Since Pedisurv is based on a voluntary participation we explored the hospital discharge data (HDD). In Belgium, the HDD are managed by the Federal Public Service of Health and are an electronic collection of anonymised records of patients admitted to all public and private hospitals. These are available with a delay of 2 years and to ensure the anonymity of HDD data, the exact number of hospitalisations is not disclosed when between 1 and 4 and mentions “< 5”. We obtained the number of hospitalisations for the period 2009–2013 with primary discharge diagnoses of HUS (by diagnosis code International Classification of Diseases, Ninth Revision, Clinical Modification (ICD-9-CM) code 283.11). Additionally, other codes were obtained to identify potentially misdiagnosed cases of HUS: acute kidney failure with acquired haemolytic anaemia and thrombocytopenia (584 and 283 and 287), thrombotic microangiopathy and intestinal infections due to other organisms (446.6 and 008) and thrombotic microangiopathy and ill-defined intestinal infections (446.6 and 009). As for the HDD with codes other than HUS (283.11), the number of cases was below 5 and therefore there were probably no misdiagnosed cases. For this reason, only the HUS cases with the code 283.11 will be used in this article.

We divided the annual number of notifications to Pedisurv and number of hospitalisations by the age-specific Belgian population for the corresponding years to obtain notification and hospitalisation rates [15]. Analyses were performed using SAS Enterprise Guide (version 5.1) and Microsoft Excel (version 12.0).

## Results

In total 110 cases of HUS were reported to the Pedisurv network during the period 2009 to 2015 with the highest number of cases in 2011 (Table 1). Not all information was available for each case of HUS (Table 2). Median age of the cases was 4.5 years (interquartile range: 2.0–7.0), male/female ratio was 0.7. Over half of the cases were notified during summer. The mean annual notification rate for Pedisurv was 0.8 per 100,000 in children < 15 years (range, 0.4–1.3) and 1.2 per 100,000 in children < 5 years (range, 0.6–2.1).

**Table 1:** Annual number of cases, notification rates and incidences of cases for Pedisurv and HDD

Year	Pedisurv: Number of cases (notification rate/10^5^) < 15 years	HDD: Number of cases (incidence/10^5^)	HDD: Number of cases (incidence/10^5^) < 5 years
		< 15 years	
2009	20 (1.1)	78 (4.3)	40 (6.4)
2010	19 (1.0)	48 (2.6)	22 (3.5)
2011	25 (1.3)	41 (2.2)	29 (4.5)
2012	9 (0.5)	58 (3.1)	20 (3.1)
2013	19 (1.0)	77 (4.1)	32 (4.9)
2014	7 (0.4)	/	/
2015	11 (0.6)	/	/
Mean	16 (0.8)	60 (3.2)	29 (4.5)

**Table 2:** Characteristics of cases reported to Pedisurv network 2009–2015

	n (%) or median (min-max) or ratio	n total
Characteristics		
Median age, years	5 (0–14)	109
age < 5 years	54 (49.5)	109
Male/female ratio	0.7	97
June-Augustus (summer)	60 (54.5)	110
illness severity		
Death	2 (2.5)	79
median days hospitalised (min-max)	10.0 (1–77)	52
T + D-	27 (33.7)	80
T-D+	3 (3.7)	80
T + D+	36 (45.0)	80
T+	63 (77.8)	81
D+	41 (50.6)	81
bloody diarrhea	59 (72.0)	82
laboratory testing^ab^		
STEC O157 isolated from stool or detected in blood	29 (26.4)	110
STEC non-O157 isolated from stool or detected in blood	10 (9.1)	110
STEC without serotyping	1 (0.9)	110

T+: transfusion, T-: no transfusion, D+: dialysis, D-: no dialysis

^a^Pedisurv data completed with NRC data. Samples were not systematically sent to the NRC

^b^One patient was co-infected with STEC O157 and STEC O103

On average, 60 (range, 41–78) HUS cases were hospitalised annually. Mean annual hospitalisation rate was 3.2 per 100,000 (range, 2.2–4.3) for children under 15 years and 4.5 (range, 3.1–6.4) for children under 5 years.

The number of cases reported to Pedisurv in comparison with the number of cases reported in HDD is not stable throughout those years: a rise is observed between 2009 and 2011 with a maximum in 2011 and is lower for 2013 and 2014 (Table 1).

### Clinical data and severity of illness

The presence or absence of diarrhoea before HUS was known for 89 patients, 79 (88.8%) of these patients had diarrhoea in the previous days and 70.6% of these patients were hospitalized following this diarrhoea. The diarrhoea was bloody in 72% of patients (Table 2). The median number of days between the onset of diarrhoea and the date of diagnosis of HUS was known for 71 patients and was 4 days (min-max: 0–19 days).

Erythrocyte transfusion was needed in 77.8% of children and dialysis in 50.6% (Table 2). Forty-five percent of patients had to have both dialysis and transfusion. The median number of days of hospitalization was known for 52 patients and was 10 days (min-max: 1–77 days). Two deaths by ischemic heart failure were reported between 2009 and 2015. Transfer to a tertiary hospital was needed for 66.6% of patients (52/78).

### Microbiologic investigations

One third of the HUS cases (35.4%, 39 cases) were confirmed positive for STEC, with a majority of STEC O157 (Table 2). Among these 39 STEC confirmed cases, 31 (28.2%) were confirmed by the NRC (29 by culture and 2 by serology).

For the 8 remaining cases that were not confirmed by the NRC, 3 STEC O157:H7, 2 STEC O157, 1 STEC O111 and 1 STEC O145 were found. These cases were confirmed at other hospital laboratories. One case was STEC confirmed without serotyping. The distribution of serotypes of STEC confirmed by the NRC and the detected virulence genes are shown in Table 3. One patient had a co-infection of STEC O157 and STEC O103. All of the STEC isolated from HUS patients possessed one or more virulence genes. Out of the 30 isolated STEC strains, 93.3% (28/30) carried the *stx2* gene, especially the *stx2a* subtype (24/28), and 96.7% possessed the *eaeA* gene (29/30). The two cases that were considered STEC positive by serology were O26 and O157 positive, respectively.

**Table 3:** Characteristics and virulence genes of STEC isolates in stool samples detected by the National Reference Centre for STEC

	Serotype	*ehxA* gene	*eaeA* gene	*stx1* gene	*stx1* subtype	*stx2* gene	*stx2* subtype
13	O157:H7	+	+	–	NA	+	*stx2a*
2	O157:H7	+	+	+	*stx1a*	+	*stx2a*
2	O157:H7	+	+	–	NA	+	*stx2a, stx2c*
1	O157:H7	+	+	–	NA	+	*stx2c*
1	O157:H7✦	+	+	–	NA	+	*stx2c*
1	O157:H-	+	+	–	NA	+	*stx2c*
1	O157:H-	+	+	+	*stx1a*	+	*stx2c*
1	O157:H-	+	+	–	NA	+	*stx2a*
1	O157:H-■	+	+	–	NA	+	*stx2a*
2	O145	+	+	–	NA	+	*stx2a*
1	O145:H-	+	+	–	NA	+	*stx2a*
1	O145:H-	+	+	+	*stx1a*	–	NA
1	O104:H4*	–	–	–	NA	+	*stx2a*
1	O103✦	+	+	+	*stx1a*	–	NA
1	O121:H19	+	+	–	NA	+	*stx2a*

Shiga toxin produced and shiga toxin genes present (+) or absent (−)*

carriage of the *aaiC* and *aggR* virulence genes typical for enteroaggregative *E. coli* (EAEC)

■ sorbitol fermenting

✦ one patient was co-infected with STEC O157:H7 and STEC O103:Hunk

*NA* not applicable, *Hunk* H-antigen type unknown

### Exposure to risk factors

The encountered risk factors during the 15 days preceding the symptoms were known for 101 cases and the most common one was beef consumption (25.7%). The intake of raw ground meat was also an important risk factor (21.8%) followed by contact with animals from a farm (13.0%) and swimming (9.0%). Consumption of unpasteurized cheese (5.9%) and unpasteurized milk (1.0%) was uncommon.

## Discussion

The overall incidence of HUS in Belgium estimated with the hospital discharge data is higher than in other European countries [4, 5, 16–19] and the United States [20]. Incidence in children < 5 years was also higher compared to other European countries. The notification rate of HUS based on the sentinel surveillance network of Pedisurv is similar to the estimates of European countries. An estimate of the incidence of HUS in Belgium in 1996 already showed similar results for children < 5 years and a lower incidence for < 15 years [10]. The incidence calculated by the hospital discharge data is probably close to the real incidence of HUS in Belgium.

The high incidence in Belgium can be explained by the methodology of this study: in Belgium, hospital discharge data are initially used by hospitals to receive state funding and because HUS is a serious condition, it is likely all cases are recorded. Moreover, all hospitals (public and private) participated to the collection of hospital discharge data. This is not the case in some other studies that used another methodology and therefore probably underestimated the incidence of HUS with only a part of the cases being collected [5, 16–20]. It is difficult to compare these rates with those of other European countries or the United States, as the number of cases fluctuated over the years, the case definition was not always the same (< 18 years for the United States) [20], and different methods were used to assess incidence. Indeed, sentinel surveillance was used in France [5], Italy [19] and the United Kingdom and Ireland [17]. In Germany and Austria in 1997–2000, a prospective multicentre study was performed and it was assumed that all patients with HUS were included [4]. Modelization from HUS notification data was used by Germany to estimate STEC HUS incidence in a recent study [16], HDD was used with mandatory notification in Norway [18] and HDD with sentinel surveillance in the US [20].

Furthermore, an American study comparing two data collection methodologies (provider-based surveillance and hospitalisation data) showed that only 49% of the HUS cases were identified by both methods [8]. Indeed, surveillance for syndromes such as HUS is challenging because detailed clinical and laboratory information is needed to validate the diagnosis.

Finally, a slight overestimation of the incidence of HUS in Belgium is possible because the ICD-9-CM 283.11 code used for hospital discharge data is not specific to HUS. There are probably aHUS among the cases reported in Belgium.

The high incidence of HUS in Belgium couldn’t be explain by a high incidence of STEC: the incidence of STEC estimated by the Belgian NRC between 2011 and 2015 was between 0.77/100,000 inhabitants (2014) to 0.94/100,000 inhabitants (2012), less than the rate calculated for Europe in 2015 (1,5/100,000 inhabitants) and less than other European countries such as in Ireland (12.9/100,000), Sweden (5.7/100,000), the Netherlands (5.1/100,000) and Denmark (3.6/100,000) [21].

Monitoring of HUS and STEC infections in Belgium is considered to be comprehensive as it is currently done through mandatory notification, the NRC and a network of laboratories monitoring STEC and the Pedisurv network for paediatric HUS. This surveillance makes it possible to meet all the objectives for the monitoring of HUS and STEC infections [9].

However, it is not yet possible to determine whether the number of cases reported via Pedisurv can be used to monitor trends in HUS cases in Belgium. By comparing the number of cases reported through Pedisurv with hospital discharge data, the proportion of cases reported via Pedisurv increased in the first 3 years and then decreased in subsequent years. This is probably due to the motivation of the paediatricians involved in the launch of this surveillance and the awareness of this disease following the outbreak of STEC O104:H4 in Germany in 2011 [22]. The next years will probably show whether a real trend can be observed within the Pedisurv network. Another limitation for this study is the underestimation of the incidence of HUS with Pedisurv because the precise coverage of the participating paediatricians is not known.

As seen in other European countries, a higher rate of HUS was observed during summer [4–6, 17–19]. This is probably due to the seasonality of STEC infections in humans, as a majority of the human cases of STEC occurred between June and September in Belgium (60% of HUS cases) [23] and in other European countries [6, 24].

The burden of disease (illness severity) is high as observed in other European countries and the United States with a high lethality rate, a long duration of hospitalization and frequentl need for dialysis or transfusion or both [4, 5, 17, 19, 20].

Some of the variables in the Pedisurv questionnaire contained missing data, which decreased the accuracy of the results, especially the outcome and the number of days of hospitalization. In addition, some questions were asked in the form of open-ended questions leading to too many different answers that did not allow an analysis of these variables, as for example history of diarrhea in family members. It was not possible to detect outbreaks with Pedisurv because all the cases were not collected with this network and because of the use of open-ended questions. The questionnaire was therefore improved in 2016 in order to reduce the number of missing data and no longer to have open-ended questions.

A clinical sample or a strain was only sent to the NRC for a part of the 110 cases of HUS notified to the Pedisurv network. Therefore, only 28.2% (31/110) were STEC confirmed by the NRC while 88.8% of cases had diarrhoea. This number is quite low and shows that peripheral laboratories need to be made aware of the need to send a clinical sample to the NRC in case of HUS. Some cases of HUS did not present diarrhoea at the time of HUS diagnosis and therefore the stools were not sent to the NRC. However it is still possible to send an rectal swab as recommended in a recent study [25] when enteropathogen identification and rapid detection is needed as is the case for HUS. The low rate of STEC positive samples could also be explained by the fact that the patient no longer excretes the bacteria in the stools at the time of the analysis [18]. In addition, some of the HUS cases in this study probably had a different aetiology than STEC infection. In this study, it is quite unlikely that atypical HUS accounts for a significant proportion of cases, as in contrast with adults who have much less risk to develop HUS after STEC infection, the latter is responsible for most HUS cases in children [7].

STEC O157:H7/H- remains the dominant cause of HUS in Belgium and other O serogroups such as STEC O145, O26 and O103 also contribute to the disease. This is quite similar in other European countries for which STEC O157:H7 were isolated in 36% of HUS cases and STEC O157:H- in 15% of the cases. STEC O145 seems to be less frequent in Europe (0.6% of cases of HUS) and other serotypes as O26:H- are on the other hand more frequent in Europe than in Belgium [6].

STEC O157 is the most circulating serogroup in Belgium (54.7% of STEC) [26] and in Europe [6]. STEC O26 is also common in Belgium (8.1% of circulating STEC) followed by O103 (3.2%) and O111 (3.1%) [26]. All this serogroups are well known to be virulent for HUS [27].

Most of de STEC strains followed Scheutz’s definition of HUS-associated *E. coli* [28] and produce *stx2*, especially *stx2a* (with or without *stx1*) and harbor *eaeA* genes. A recent study from the NRC for STEC in Belgium confirmed this observation and showed that the most important risks factors for HUS development were the presence of the *stx2* gene, especially *stx2a*, and the *eaeA* gene [29]. Similar studies were conducted in other European countries as Norway and showed the same results [30]. In Europe, most of the HUS stains in 2010–2012 were also *stx2* positive [6].

One case of HUS was caused by a *stx2a* positive STEC O104:H4 that did not carry the *ehxA* and *eaeA* genes, but did possess virulence genes of enteroaggregative *E. coli*. This strain was observed after travel in a Mediterranean country (Turkey) and was not linked with the German outbreak of 2011 [14].

Another case of HUS was caused by a sorbitol fermenting O157. This type of strain is known to be more virulent, but is very rare in Belgium [26]. It is however rather frequent in Germany where it is found in 10% of cases of HUS [4] and also regularly causes outbreaks [31, 32].

Serogroup O121 is also rather rare in Belgium, but is known to be part of the ‘pathogenic gang of six’; the six most virulent O serogroups [27].

Beef and minced meat consumption are the most frequent reported risk exposures in this study, which is also described in the literature [33]. However, these are not confirmed sources of infection and given the large number of foods that can cause contamination, it is often difficult to investigate and find this source of infection, especially in case of isolated cases.

## Conclusion

As observed in 1996, the incidence of HUS in Belgium is still high compared to other European countries.

HUS is a severe condition and its surveillance, although less effective than monitoring STEC to detect the emergence of outbreaks, is important in order to better understand the more pathogenic STEC strains. In this context, efforts are still needed to send strains and samples (especially rectal swabs, to the NRC in case of HUS.

It would be interesting to compare the number of cases notified to Pedisurv with hospital discharge data in future years to see if a trend can be observed through Pedisurv.

## Abbreviation

aHUS: Atypical Haemolytic Uremic Syndrome
HDD: Hospital Discharge Data
HUS: Haemolytic Uremic Syndrome
NRC: National Reference Centre
STEC-HUS: Haemolytic Uremic Syndrome caused by Shiga-toxin *Escherichia coli* infection
